# Supplementary material for: The interactome of intact mitochondria by cross-linking mass spectrometry provides evidence for coexisting respiratory supercomplexes
Source: Mol Cell Proteomics. 2017 Dec 8;17(2):216–32. doi: 10.1074/mcp.RA117.000470 (PMC5795388; doi:10.1074/mcp.RA117.000470)
Supplement: Supplemental Data [file supp_RA117.000470_133922_0_supp_23978_fzffwf.zip › spectra_annotation/mito_spectra_annotation/417-1-4-1-12-1.pdf]

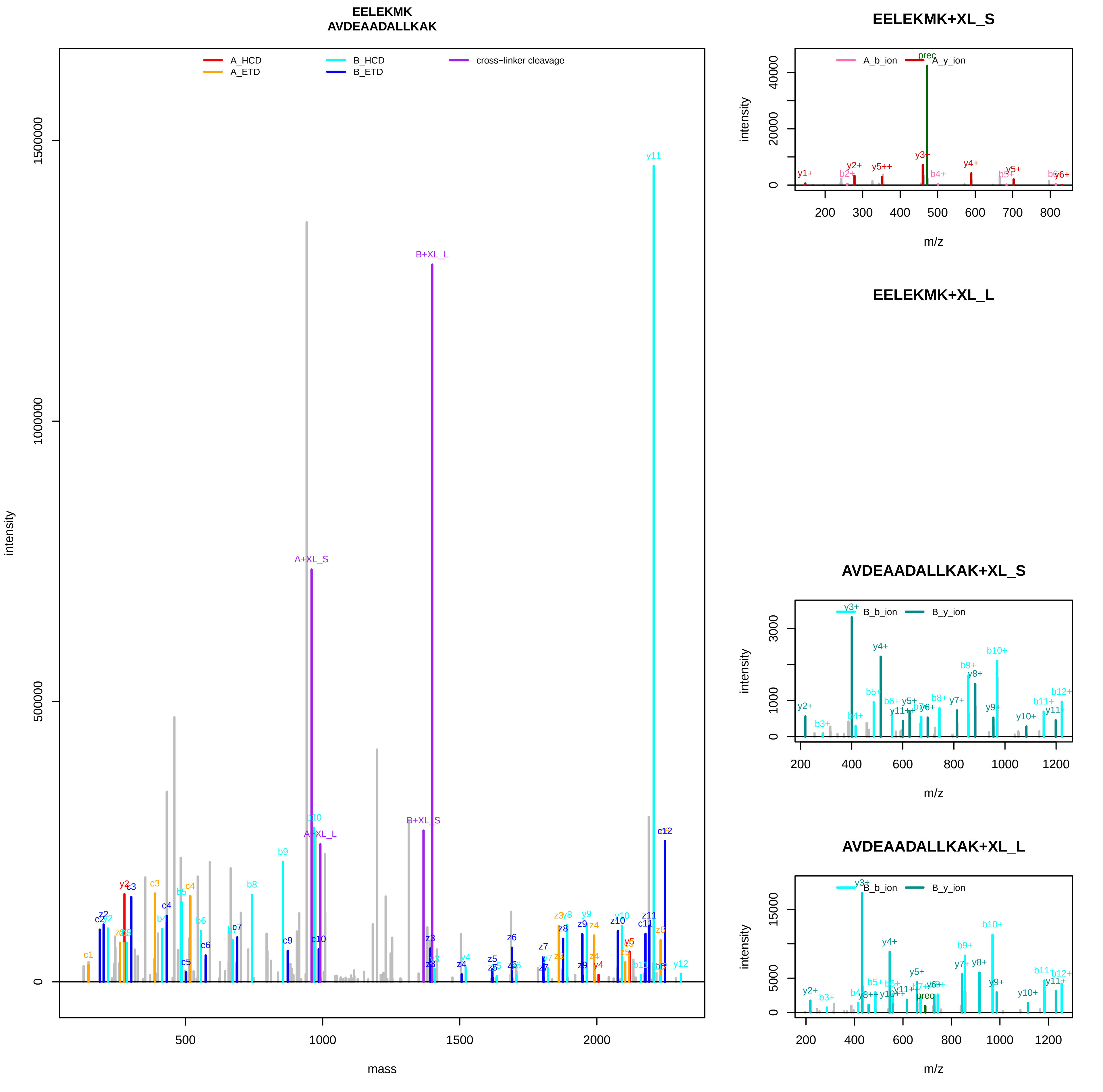

EELEKMK
AVDEAADALLKAK
A_HCD
A_ETD
B_HCD
B_ETD
cross−linker cleavage
intensity
0
500000
1000000
1500000
mass
500
1000
1500
2000
y11
B+XL_L
A+XL_S
B+XL_S
A+XL_L
b10
b9
b8
c12
EELEKMK+XL_S
A_b_ion
A_y_ion
prec
intensity
0
20000
40000
m/z
200
300
400
500
600
700
800
EELEKMK+XL_L
AVDEAADALLKAK+XL_S
B_b_ion
B_y_ion
intensity
0
1000
3000
m/z
200
400
600
800
1000
1200
AVDEAADALLKAK+XL_L
B_b_ion
B_y_ion
intensity
0
5000
15000
m/z
200
400
600
800
1000
1200
